# A Rare Case of a Right Atrial Thrombus

**DOI:** 10.7759/cureus.80543

**Published:** 2025-03-13

**Authors:** Zeyn Mahomed, Tamar Gerges, Waunita Naidoo, Shiraz Harypursat, Nabeelah Nalla, Dilkash Harryprasadh, Craig Beringer, Deidre Hoffman, Mohammad Ghanty, Sumika Singh, Peter Beskyd

**Affiliations:** 1 Emergency Medicine, University of the Witwatersrand, Johannesburg, Johannesburg, ZAF; 2 Emergency, Chris Hani Baragwanath Academic Hospital, Johannesburg, ZAF; 3 Internal Medicine, Chris Hani Baragwanath Academic Hospital, Johannesburg, ZAF; 4 Emergency Medicine, Chris Hani Baragwanath Academic Hospital, Johannesburg, ZAF

**Keywords:** hiv diseases, intravenous thrombolysis, narrow complex tachycardia, point-of-care ultrasound, right atrial thrombus

## Abstract

Right heart thrombi are a rare entity and are most often a result of deep vein thrombosis or structural heart disease. Right heart thrombi are frequently associated with pulmonary emboli and are often undiagnosed, leading to significant morbidity and mortality. The use of bedside ultrasound has made it far easier to identify these right heart thrombi or clots in transit. Early identification leads to early management and also aids the clinician in distinguishing the presentation from the many other causes of chest pain and shortness of breath.

The authors present a case of a right atrial thrombus in an immunocompromised adult male patient. In this case report, the authors describe the investigations that led to the rapid identification of the right heart thrombus. Therapeutic options traditionally include surgical embolectomy, thrombolysis, anticoagulation, and percutaneous retrieval techniques. The authors were based in a resource-limited setting and will discuss the decision-making process that ensued.

## Introduction

Following the European Cooperative Study on the clinical significance of right heart thrombi in 1989, the European Working Group on Echocardiography identified three types of right atrial thrombi [1,2]. The distinction between the three types was based on the morphology, etiology, and clinical significance of the thrombus [3,4].

Type A thrombi are described as highly mobile and serpiginous. They show a strong association with deep vein thrombosis (DVT), and it is thus postulated that they originate in peripheral veins. They were found to be associated with pulmonary embolism in all cases, a correlation that has been demonstrated in multiple studies [5,6]. Type B thrombi are immobile, adherent to the cardiac chamber wall, and, unlike Type A thrombi, form in the presence of underlying cardiac abnormalities. Type C thrombi are highly mobile, but they are not worm-shaped like the Type A thrombi. Type C therefore has mixed characteristics.

The authors present a rare case of a Type A right atrial thrombus. This thrombus was identified in a patient who has a background history of dilated cardiomyopathy. Although it is highly likely that the patient developed a pulmonary embolus while in the emergency department (ED), the development of a hemodynamically unstable pulmonary embolism was averted.

## Case presentation

A 52-year-old HIV-positive man with a CD4 count of 200 presented to the ED with complaints of worsening effort tolerance (New York Heart Association (NYHA) Classification 3), orthopnea, non-productive cough, and loss of appetite. He denied any history of palpitations, chest pain, dizziness, or syncope.

One week prior, he had been discharged from the hospital. He had been admitted to a general medical ward for acute decompensated heart failure. During his five-day admission, he was initiated on antifailure treatment and then discharged on oral hypoglycemic agents as a newly diagnosed type 2 diabetic. His NYHA effort tolerance when leaving the hospital was a grade 1. Upon discharge, the patient was reminded to continue with his anti-retroviral therapy.

**Figure 1:**
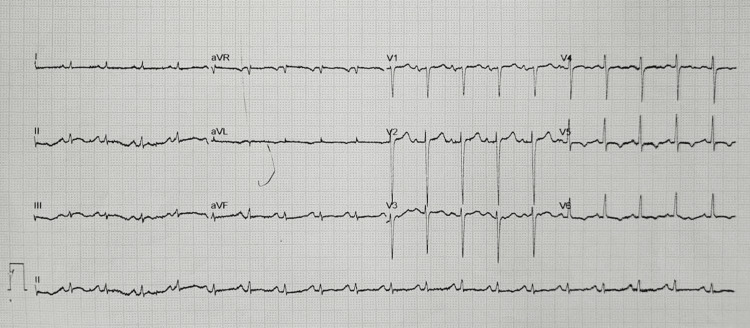
Initial 12 lead ECG obtained in triage.

On arrival at the ED, he was noted to be in respiratory distress with a saturation of 88% on pulse oximetry. He was placed on nasal prong oxygen at 4 liters per minute, and his respiratory distress improved. The patient's blood pressure on arrival was 146/94. The initial ECG revealed sinus tachycardia, a normal axis, and P pulmonale (Figure 1). No acute ischemic changes were noted. The venous blood gas showed a lactate of 2.5, pH of 7.44, PCO_2 _of 39 mmHg, base excess of 2.3 mmol/L, and HCO_3_ of 26.5 mmol/L.

On examination, auscultation of the chest revealed bilateral basal crepitations. The cardiovascular exam revealed grade 2 bipedal pitting edema and elevated jugular venous pressure. Both calves were soft and non-tender. All other systems were unremarkable.

**Figure 2:**
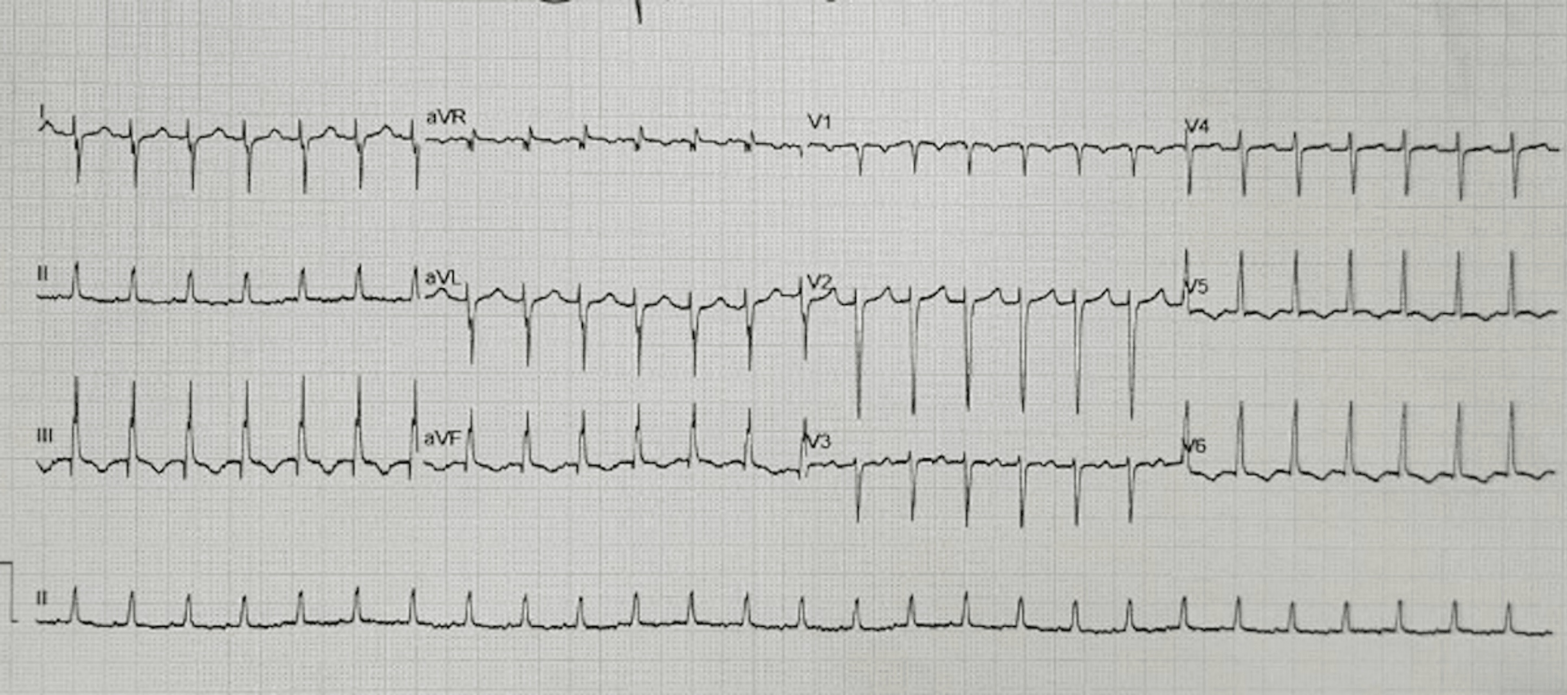
Repeat 12 lead ECG showing the development of a regular narrow complex tachycardia.

It was at this point in the examination that the patient developed tachycardia with heart rates ranging from 163 to 171. A repeat ECG (Figure 2) showed a narrow complex tachycardia with no discernible P waves. During this episode, the patient remained relatively stable with a lower blood pressure of 108/63 and no marked change with regard to respiratory effort. The patient denied any symptoms relating to his tachycardia, such as dizziness, palpitations, or chest pain.

**Figure 3:**
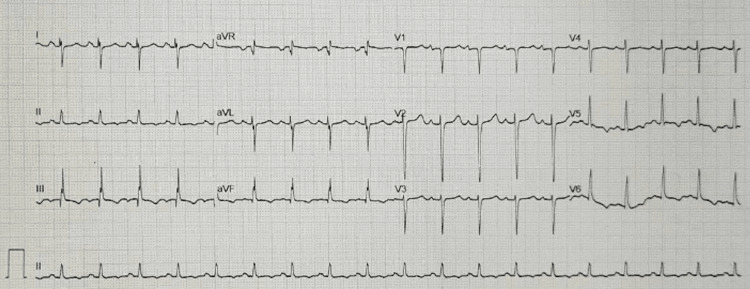
ECG post modified Valsalva maneuver.

The patient was subsequently moved to the resuscitation area, and a modified Valsalva maneuver was performed, after which the rhythm reverted to sinus tachycardia at 114 bpm. The repeat ECG resembled his initial triage ECG, now with T-wave inversion in the inferior leads (Figure 3). Also of note, in the repeat ECG, there was a new right-sided axis deviation, as well as an S wave in lead I, a Q wave in lead III, and a T wave inversion in the lateral leads. The repeat ECG suggests the development of a pulmonary embolism.

**Figure 4:**
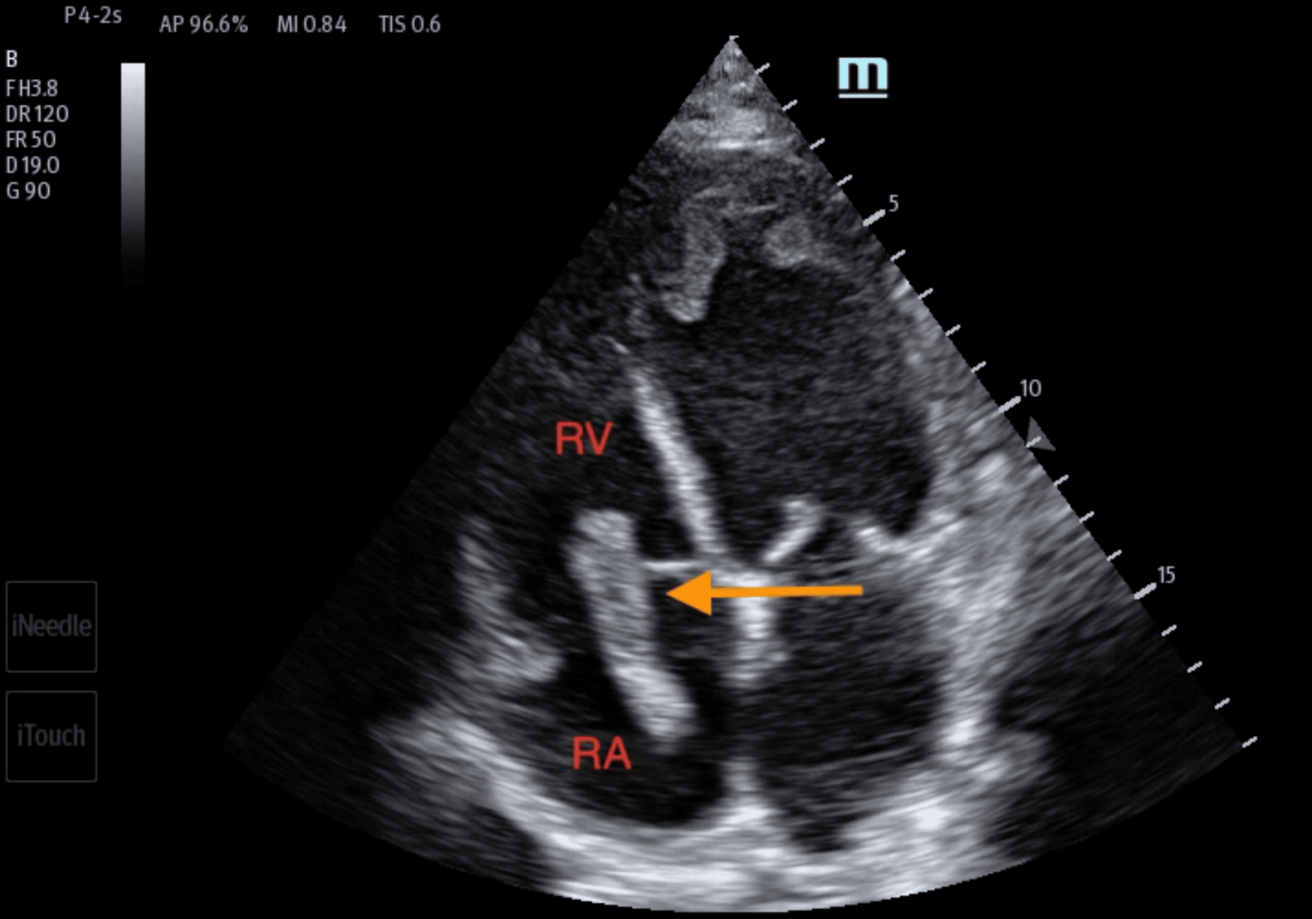
Transthoracic echocardiogram imaging of the apical four-chamber view showing long, serpiginous, and mobile thrombus (orange arrow) in the right heart chambers. RA: right atrium; RV: right ventricle

**Figure 5:**
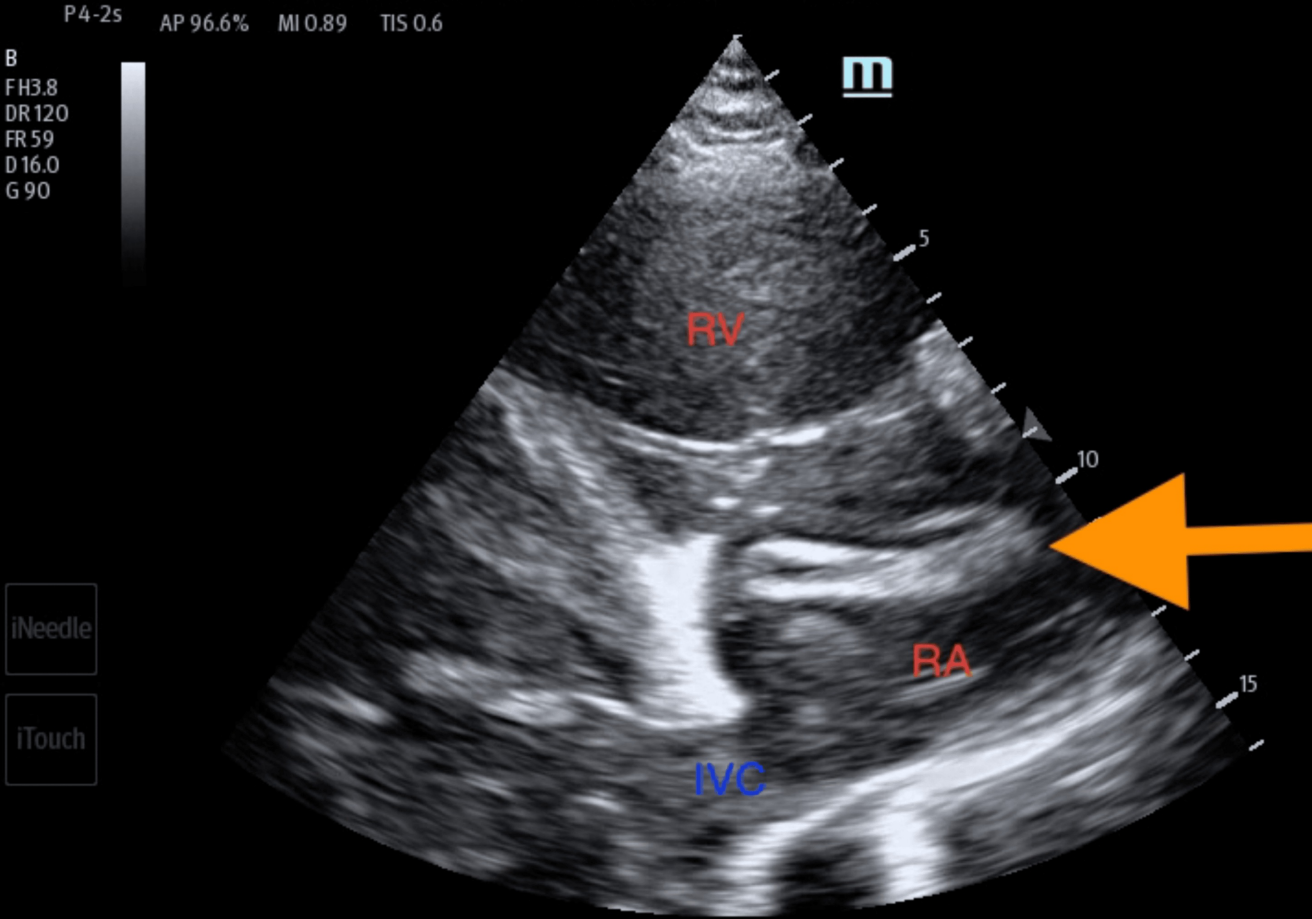
Transthoracic echocardiogram imaging of the right ventricular inflow tract illustrating thrombus in the right atrium (orange arrow). RA: right atrium; RV: right ventricle; IVC: inferior vena cava

**Figure 6:**
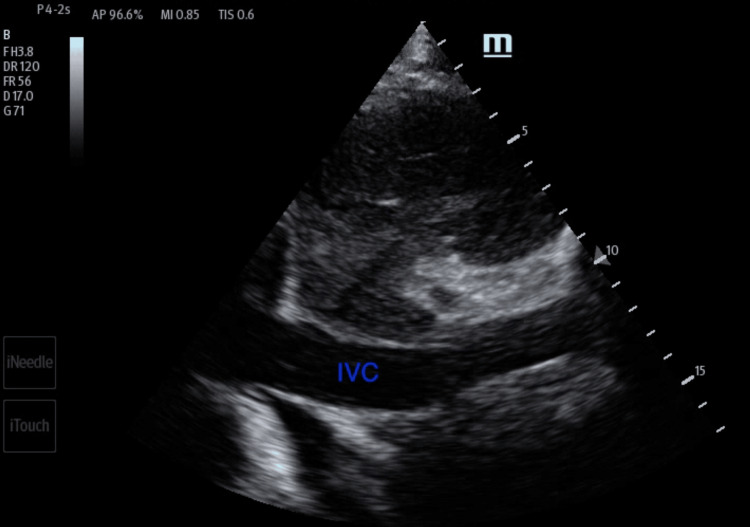
Distended inferior vena cava visualized on transthoracic echocardiogram. IVC: inferior vena cava

Any patient presenting to an ED with shortness of breath can harbor a wide range of differential diagnoses. Point-of-care ultrasound can help readily identify a potential cause and help direct the management of the patient. A point-of-care ultrasound examination of the heart revealed four-chamber dilatation with globally decreased contractility and a markedly reduced ejection fraction. A serpiginous clot in transit was noted in the right atrium and appeared at times to traverse the tricuspid valve (Figures 4-5). There were no features of overt right heart strain, as there was an absent D-sign and no McConnell's sign. The inferior vena cava, though enlarged, varied with respiration (Figure 6). A three-point limited compression ultrasound was performed, which showed bilaterally compressible femoral and popliteal veins with no evidence of DVT. This thus does not completely exclude the existence of a DVT, as point-of-care ultrasound is operator-dependent and pelvic DVTs cannot be excluded.

**Figure 7:**
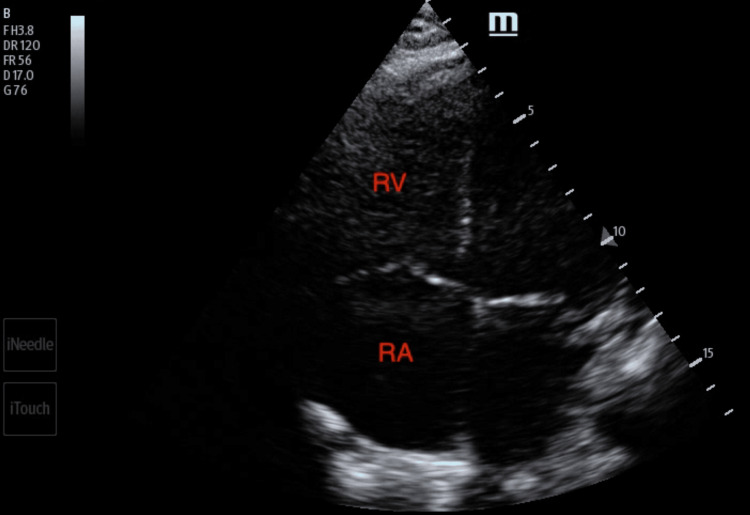
Transthoracic echocardiogram done in the apical four-chamber view post-thrombolysis, showing complete resolution of the thrombus previously visualized in the right atrium. RA: right atrium; RV: right ventricle

A decision was made to administer a thrombolytic. The patient received 10 mg of alteplase (tPA) as a bolus, followed by an infusion of 40 mg over two hours. The patient remained stable throughout, and upon completion of thrombolysis, his repeat bedside echocardiogram showed complete resolution of the clot (Figure 7). The repeat ultrasound was performed by the same operator using the same machine and was performed approximately 45 minutes after the thrombolytic infusion had been completed. Following an uneventful period of observation in the ED post-thrombolysis, he was admitted to a general medical ward. Due to the limited availability of high-care and intensive-care unit beds, the patient was admitted to a standard medical ward.

It would have been ideal to have a computed tomography pulmonary angiogram (CTPA) performed in this case; however, due to the resource limitations and the information provided by the point-of-care ultrasound, a CTPA was not performed during the patient's in-hospital stay. The patient was commenced on a direct oral anticoagulant with cardiology outpatient follow-up.

## Discussion

With the increased use of antiretroviral drugs, people with HIV live longer. As such, there is a shift in the clinical sequelae of the disease from those associated with acute opportunistic infections to long-term non-communicable diseases that commonly affect older generations. One organ system frequently affected by this shift in the disease profile is the cardiovascular system. It is anticipated that by the year 2030, 73% of people infected with HIV will be ≥50 years old, and 78% will be affected by cardiovascular disease [7]. With the increasing use of highly active antiretroviral therapy, cardiac manifestations are trending away from the previously seen dilated cardiomyopathy with profound left ventricular systolic dysfunction to cardiac pathology with diastolic dysfunction and ischemic heart disease [8]. In low- and middle-income countries, however, HIV-associated cardiomyopathy remains the most common manifestation of HIV-associated cardiovascular disease.

Regardless of the cause of this patient’s cardiomyopathy, the incidence of DVT in congestive cardiac failure (CCF) is high. Proven DVTs occur in 10% to 20% of hospitalized CCF patients. This is attributable to a variety of abnormalities in Virchow’s triad, as well as an associated chronic inflammatory state. This, together with severe left ventricular dysfunction and clinical severity measured by an NYHA class III/IV, enhances the risk for venous thromboembolism (VTE). Furthermore, VTE phenomena are 2 to 10 times more likely in patients with HIV, and those with low CD4 counts are at increased risk [9]. This patient had many risk factors predisposing him to the development of a Type A right heart thrombus.

Type A thrombi are invariably associated with pulmonary embolism and portend a poorer prognosis [4,5]. Patients with pulmonary embolism in the context of a right heart thrombus have been shown to have a shorter duration of symptoms, lower systolic blood pressures, right ventricular hypokinesis, and CCF [6]. Type A thrombus-related mortality is 42%, compared to 4% in patients with Type B right heart thrombi [4]. This patient remained normotensive throughout his presentation and had no echocardiographic features suggestive of a clinically significant pulmonary embolus; however, there were ECG changes suggesting that the patient had developed a pulmonary embolism whilst in the ED.

Treatment options for right heart thrombi include anticoagulation, thrombolysis, or surgical embolectomy. There is currently no evidence to support one treatment option over the other, and there are no standardized guidelines for the management of right heart thrombi. Management decisions are made on a case-by-case basis. The decision to thrombolyze this patient with half-dose tPA was made based on evidence that “safe dose” thrombolysis (half the standard dose) is both safe and effective in treating patients with moderate PE [10]. "Safe dose” thrombolysis was also shown to significantly reduce the risk of pulmonary hypertension and recurrent PE (compared to anticoagulation alone). It was also associated with no bleeding events.

Although there were no significant echocardiographic findings suggesting an acute pulmonary embolus, and even though the patient remained hemodynamically stable, the size of the clot in transit, as well as the ECG changes in Figure 3, swayed the physicians into making the decision to proceed with thrombolysis. A pulmonary embolism was not confirmed on CTPA, as decision-making was based solely on point-of-care ultrasound findings, hemodynamic parameters, and clinical acumen. In a resource-limited environment, decisions often have to be made based on these criteria alone.

## Conclusions

Right heart thrombi are a medical emergency requiring prompt diagnosis and intervention. Delayed identification and management lead to significant morbidity and mortality. This case underscores the importance of bedside ultrasound in the rapid diagnosis of right heart thrombi, particularly in resource-limited settings. Furthermore, in resource-limited settings, thrombolysis may be the only viable option despite inherent risks.
